# Plasma Concentration of Prolactin, Testosterone Might Be Associated with Brain Response to Visual Erotic Stimuli in Healthy Heterosexual Males

**DOI:** 10.4306/pi.2009.6.3.194

**Published:** 2009-08-12

**Authors:** Younghee Seo, Bumseok Jeong, Ji-Woong Kim, Jeewook Choi

**Affiliations:** 1 Department of Psychiatry, Eulji University College of Medicine, Daejeon, Korea.; 2 Department of Psychiatry, College of Medical Science, Kongyang University, Daejeon, Korea.; 3 Department of Psychiatry, Daejeon St. Mary's Hospital, The Catholic University of Korea College of Medicine, Daejeon, Korea.

**Keywords:** Prolactin, Testosterone, Sexual behavior, Magnetic resonance imaging, Dopamine

## Abstract

**Objective:**

Many studies have showed that excess or lack of sexual hormones, such as prolactin and testosterone, induced the sexual dysfunction in humans. Little, however, is known about the role of sexual hormones showing normal range in, especially, the basal state unexposed to any sexual stimulation. We hypothesized sexual hormones in the basal state may affect sexual behavior.

**Methods:**

We investigated the association of the sexual hormones level in the basal hormonal state before visual sexual stimulation with the sexual response-related brain activity during the stimulation. Twelve heterosexual men were recorded the functional MRI signals of their brain activation elicited by passive viewing erotic (ERO), happy-faced (HA) couple, food and nature pictures. Both plasma prolacitn and testosterone concentrations were measured before functional MR scanning. A voxel wise regression analyses were performed to investigate the relationship between the concentration of sexual hormones in basal state and brain activity elicited by ERO minus HA, not food minus nature, contrast.

**Results:**

The plasma concentration of prolactin in basal state showed positive association with the activity of the brain involving cognitive component of sexual behavior including the left middle frontal gyrus, paracingulate/superior frontal/anterior cingulate gyri, bilateral parietal lobule, right angular, bilateral precuneus and right cerebellum. Testosterone in basal state was positively associated with the brain activity of the bilateral supplementary motor area which related with motivational component of sexual behavior.

**Conclusion:**

Our results suggested sexual hormones in basal state may have their specific target regions or network associated with sexual response.

## Introduction

Human sexual experience is a multifactorial response comprising of physiological, psychological and cultural factors.^1^-^4^ The endocrine system is one of major physiologic factors associated with human sexual response, and several hormones, produced by endocrine system, such as prolactin, oxytocin, and testosterone have been related with sexual behavior.^5^

Prolactin and oxytocin participate in the regulation of animal and human sexual behavior,^5^-^7^ for example, their plasma concentration is increased immediately after orgasm in human, especially prolactin.^7^ Acute alterations of prolactin by pharmacological manipulation in healthy men might affect sexual drive, ejaculation latency, appetitive and consummatory sexual behavior.^8^ Chronic hyperprolactinemia also causes a significant reduction of sexual desire, erectile dysfunction, and infertility in men and women.^9^,^10^ Hypoprolactinemia also induce the several problems, such as menstrual disorder, infertility, and sexual dysfunction.^11^,^12^ The plasma concentration of testosterone, an important hormone to sex differentiation and sexual function, is correlated with higher levels of penile response and sexual motivation in males.^13^-^15^ Testosterone deficiency also induces the erectile dysfunction, decreased sexual interest.^16^ Previous studies^17^ have been focusing on the effect of phasic release or abnormal state of sex hormones, such as an acute alteration of hormonal level followed by an orgasm, pharmacological manipulation or the maintenance of abnormal concentration of sexual hormone such as testosterone deficiency, hyper- or hypo-prolactinemia, on sexual behavior.

These reports suggested that sexual behavior might be associated with sexual hormones and be changed by abnormally low or high level of sexual hormones. Little is, however, known about the role of sexual hormones in basal, not phasic release, state in individuals with normal level to sexual behavior. Interestingly, one report showed that endogenous, that is basal, testosterone level, was strongly correlated to sexual motivation on sexual behavior.^18^ We also had an interest in the role of sexual hormones, prolactin and testosterone, in basal state, though their levels varied in a pulsatile and diurnal manner. In this study, we especially focused on the relationship between the plasma concentration of sexual hormones in basal state before sexual stimulation and the sexual stimuli-induced brain activity. Questions raised herein were where sexual hormones may affect in brain and whether each sexual hormone have specific target regions or share them.

Functional neuroimaging could be a potent research tool to investigate the effect of the sexual hormones on brain function. Development of neuroimaging techniques, such as positron emission tomography (PET) or functional magnetic resonance imaging (fMRI), allow the investigation of the neuropsychologic components of sexual arousal by studying brain activation during different phases of sexual arousal. Previous PET^19^-^21^ or fMRI^22^-^27^ studies have demonstrated that male sexual arousal is associated with the activation of several brain areas, including limbic (hypothalamus, hippocampus and amygdala) and paralimbic regions (anterior cingulate gyrus, frontal lobe, parietal lobe, and insula), associative cortices (inferior temporal and occipital cortices), other subcortical and cortical sensory relays (thalamus and SII), midbrain, and cerebellum.

Considering these multi-regional results and their functional diversity, it was proposed that the neurobehavioral model of the brain processes related to human sexual arousal composed of cognitive, emotional, motivational, and autonomic components.^20^ According to the previous studies, the cognitive component comprises a process of appraisal whether stimuli are valued as sexual incentives, increased attention to stimuli, and motor imagery related to sexual behavior. The emotional component implies the hedonic feelings of sexual arousal, while motivational component relates to processes that direct sexual behavior to satisfy a sexual arousal. Finally, the autonomic component includes various physiological responses (e.g., cardiovascular, respiratory, and genital) of the individual to sexual behavior.

Based on abovementioned studies of hormone and neuroimaging, we supposed that sexual hormones in basal state may have a specific role to sexual behavior and their effect to functional component in brain may be found out by functional neuroimaging. Here, we performed the regression analysis to investigate the relationship between the plasma concentration of sexual hormone, testosterone and prolactin, measured before visual erotic stimulation (basal state of hormonal level), and sexual response-related brain activity during visual sexual stimulation.

## Methods

### Subjects

The enrollment for healthy heterosexual males was advertised through the message board in Eulji University Hospital, Daejeon, Korea. All applicants were asked about their sexual activities and sexual function including erection and ejaculation within the past week. Twelve heterosexual male subjects without any sexual dysfunction (age=30.5±6.6 years) were enrolled for the current study. Subjects were interviewed and were screened to exclude any possible psychiatric diseases by a psychiatrist using DSM-IV criteria. No subjects had an Axis-I psychiatric disorder including sexual related and eating disorder. This study was approved by the Eulji University Hosptial's Institutional Review Board. All subjects gave written informed consent prior to participation in the study. Subjects were asked to prohibit from their sexual activities such as masturbation or sexual intercourse for one day before fMR scanning. At the day of fMR scanning, subjects were asked about their sexual activities and sexual function including sexual desire, ability to have an erection, maintenance of erection as long as necessary to have intercourse, ejaculation failure within the past week (Table 1).

### Visual stimulation with pictures of nature, food, happy-faced or erotic couple

Although, in the present study, a relationship between the normal or abnormal plasma concentration of prolactin or testosterone and brain activation in response to erotic stimuli would be demonstrated, it might be unclear whether the hormonal relationship is stimuli-specific or not. Thus sexually neutral conditions involving pictures of nature (tree, stones, flowers, shrubs) and food (ice cream, oriental noodle, chocolate, spaghetti) were used in current study to record brain activity due to another drive, appetite. In addition, to control for male-female couple context in the erotic condition (ERO), pictures of smiling couples in non-erotic situations were also added as another experimental condition. Since the effect of erotic stimulation could be sustained over longer period of time, three ERO blocks were following three happy couple condition (HA) blocks. Three food blocks were also following three nature blocks.

The order in which conditions were presented within each experimental run was follows: nature, food, HA and ERO conditions. Fixation blocks were following each condition block. Each run was preceded by 12 seconds of dummy scans, followed by the fixation block. Each block lasted 24 seconds. Each picture was showed every 6 seconds and 4 pictures were presented in each block. All pictures of both erotic couple and nature were selected from International Affective Picture System (IAPS).^28^ Pictures of happy-faced couple were selected from both IAPS and were produced by the authors in the present study.

Subjects were requested to have meal as much as they felt full. MR scan were acquired between 10 am and 12 pm or between p.m. 2 and p.m. 5:30. Subjects were not provided any information about pictures presented before fMR scan and were passively viewing pictures during scan.

### Measure of hormone plasma concentration

To investigate the relationship of sexual hormone with brain activation, both plasma prolactin and plasma testosterone concentrations were measured 30 minutes before fMR scan. Both hormones concentration was analyzed by the chemiluminescence immunoassay (CLIA). Mean prolactin and testosterone levels were 8.6 ng/mL (SD=3.9, range=4.6-17.4, normal range=2.1-17.7) and 372.7 ng/dL (SD=82.9, range=242.0-490.9, normal range=241-827), respectively.

### Intensity of subjective feelings for pictures: comparison among each condition and relationship with scan time

After fMR scan, each subject was asked to measure both his subjective intensity of sexual desire to erotic pictures and that of food craving to food pictures presented during fMRI scan with Likert scale (1-8, 8 being the highest desire). Two-factor (2 potency level, 2 drives) repeated measured analysis of variance (ANOVA) was performed to explore the main effect of potency (food minus nature or ERO minus HA conditions) and of drive (sexual minus craving), and the potency by drive interaction. The diurnal variation, possible confounding factor for investigating inter-subject difference, of testosterone was previously reported^29^,^30^ as possibly affecting brain activation to visual erotic stimulation. Thus, nonparametric Kendall correlation analyses were performed among the plasma concentration of sexual hormones, the time interval from midnight to the sampling time and the intensity of subjective feeling to erotic pictures.

### MRI data acquisition

All subjects underwent MRI procedures on 3.0-T whole body MRI Echospeed system (ISOL, Korea). Prior to functional acquisitions, a high-resolution structural MRI examination [TE=5.7 ms, TR=10 ms, field of view (FOV)=220 mm, matrix size=256×256, slice thickness=1.5 mm, magnetization-prepared rapid acquisition with gradient echo (MPRAGE) sagittal slices] was performed for each patient in order to exclude any potential brain abnormalities. We collected the total of 135 EPI scans of the blood oxygen level-dependent responses (TE=35 ms, TR=2.4 s, FOV=192×220 mm, flip angle=70°, 5 mm thick, 2 mm gap, 64×64 matrix, 20 axial slices) and also took in-plane T1-weighted anatomical data (TE=16 ms, TR=2,800 ms, FOV=192×220 mm, matrix size=192×256, flip angle=60°, slice thickness=5 mm, 2 mm gap, 20 axial slices) for each participant.

### Processing and analysis of functional magnetic resonance imaging data

#### Acquisition and Preprocessing

fMRI data was processed and analyzed with fMRI Expert Analysis Tool (FEAT) of FSL software (http://www.fmrib.ox.ac.uk/fsl/feat5/index.html). The first 5 scans were discarded. The remaining 130 images were spatially realigned using rigid-body transformation and underwent slice timing correction.

Next, a brain mask from the first volume in fMR data was created for getting rid of signals outside of brain in each subject. To reduce noise without reducing valid activation, spatial smoothing was performed using 5 mm full width at half maximum (FWHM). fMR images were filtered with 144 seconds high pass filter. Prewhitening, removal of serial correlations, was performed to make the statistics valid and maximally efficient.

#### Statistical Analyses for Preprocessed Functional Magnetic Resonance Imaging Data

The general linear model was used for linear combination of the modeled response to visual stimulation of pictures of food, nature, ERO and HA conditions. Activation maps for the contrasts including food minus nature, ERO minus HA were constructed separately for each subject using mixed effect analysis. The resulting Z statistic image in each subject was entered to mixed effect analysis for group level regression analysis to show which clusters of voxels above 2.3 of Z value were activated at a significance level of p<0.05. Activations identified at a spatial extent of at least 10 voxels.

Before group analysis, fMRI data were registered to T1 weighted structural image with translation and to high-resolution structural image with linear transformation of 6 degreesof-freedom (FMRIB's Linear Image Registration Tool^31^, FLIRT) and finally to standard space using nonlinear registration (FMRIB's Nonlinear Image Registration Tool, FNIRT; http://www.fmrib.ox.ac.uk/fsl/fnirt/index.html).

We performed correlation analysis between the change of brain activity to ERO minus HA and the subjective sexual desire to erotic pictures presented during fMR scan. We also performed multiple regression analysis to test our hypothesis that plasma concentration of hormones including prolactin and testosterone in basal state (before erotic stimulation) may affect the change of brain activity to ERO minus HA or to food minus nature contrasts, adjusting for age, the time interval from midnight to the sampling time.

We constructed regression map for each set of two contrasts using mixed effect analysis and defined the statistically significant clusters of voxels above 2.3 of Z value at a significance level of p<0.05 with a spatial extent of at least 10 voxels.

All statistical inference method for fMR data was used nonparametric, not parametric, method.

## Results

### Intensity of subjective feeling to pictures presented and its relationship with scan time and the level of sexual hormones before visual stimulation

The score of subjective sexual desire for the presented erotic pictures and that of the happy-faced couple pictures were 5.5 (SD=2.3, range=1-8) and 2.2 (SD=1.8, range=1-7), respectively. The scores of subjective food craving for the presented food pictures and that for the pictures of nature were 4.5 (SD=2.2, range=2-8) and 1.8 (SD=0.9, range=1-3), respectively. Two-factor repeated measured ANOVA revealed the significant main effect of the intensity of derive (F=26.26, p=0.001) however neither the main effect of the nature of drive (F=4.39, p=0.066) nor interaction (F=1.50, p=0.252) was found. Thus, pictures of our paradigm evoked drive-specific desire. Nonparametric Kendall correlation analyses showed that scan time had no significant relationship with the plasma concentration of sexual hormone (testosterone: tau_b=-0.10, p=0.78; prolactin: tau_b=0.39, p=0.26) or subjective sexual desire to erotic pictures (tau_b=-0.59, p=0.07). Subjective sexual desire to erotic pictures had no significant correlation with neither testosterone (tau_b=0.27, p=0.45) nor prolactin (tau_b=-0.53, p=0.12).

### Functional magnetic resonance imaging

**FIGURE 1:**
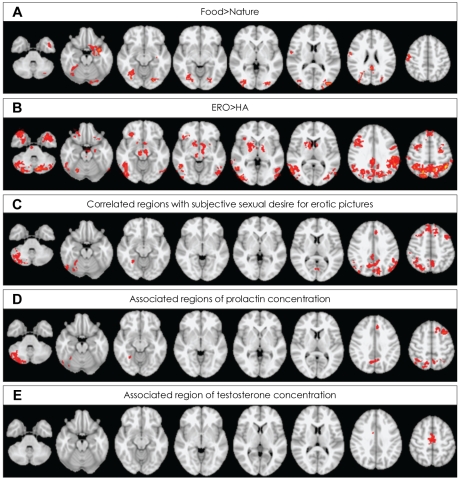
A: Regions activated for food minus nature conditions. B: Regions activated for erotic (ERO) minus happy-faced (HA) people conditions. C: Regions correlated with subjective feeling for erotic picture. D: Regions associated with plasma concentration of prolactin in basal state. E: Regions associated with plasma concentration of testosterone in basal state. Activations identified at cluster-level significance of p<0.05 for a spatial extent of at least 10 voxels.

Mean time interval from midnight to fMR scan was 816.4 (SD=148.2) minutes. When contrasting food and nature conditions, healthy male subjects demonstrated (p<0.05 at cluster level) activation in the right pre- and post-central gyrus, the bilateral posterior cingulate gyri, the bilateral supplementary motor cortices/left superior frontal gyrus, the left frontal orbital cortex, bilateral lateral occipital cortices, the bilateral occipital fusiform gyri, left temporal pole, the left parahippocampal gyrus and left hippocampal-amygdalar complex (Figure 1A). No region was more activated in nature, compared with food conditions.

When contrasting ERO and HA conditions, healthy male subjects demonstrated (p<0.05 at cluster level) activation in the bilateral middle frontal gyri, the bilateral superior frontal gyri, the bilateral precentral gyri, the bilateral frontal pole, the bilateral parieto-occipital regions consisting the left superior parietal lobule, the bilateral supramarginal gyri, the bilateral lateral occipital cortices, the left temporo-occipital junctions, the bilateral temporal poles, the left inferior temporal gyri, the bilateral parahippocampal gyri, the left hippocampal-amygdalar complex, the right nucleus accumbens/putamen, the bilateral globus pallidum, the bilateral caudate, the left thalamus, the right insula, the right midbrain, the bilateral pons and the bilateral cerebellum (Figure 1B, supplemental Table 1). No region was more activated in HA, compared with ERO conditions.

### Correlation between brain activity and subjective sexual desire to erotic pictures

The subjective sexual desire for erotic pictures showed statistically significant positive correlation with brain activity of the bilateral middle and superior frontal gyri, the left paracingulate gyrus, the right lateral occipital cortex and the cerebellum (Table 2, Figure 1C).

### Association of prolactin and testosterone plasma concentration measured before visual stimulation with the signal change of brain activity

Each sexual hormone level was significantly associated with the signal change to the contrast of ERO minus HA in different brain areas. The concentration of plasma prolactin measured before visual stimulation (basal state) showed statistically significant positive association with the brain activity of several regions including the left middle frontal gyrus, paracingulate/superior frontal/anterior cingulate gyri, the bilateral parietal lobule, the right angular, the bilateral precuneus and the right cerebellum (Table 2, Figure 1D). The concentration of plasma testosterone in basal state showed statistically significant positive association with the brain activity of the bilateral supplementary motor area (SMA)(Table 2, Figure 1E). However, no brain region showed negative association with the level of sexual hormones.

Regression analysis showed no significant association of sexual hormones' plasma concentration with the brain signal change to the contrast of food minus nature.

## Discussion

We focused on neuropsychologic aspect of sexual hormone, and investigated to the association of sexual hormones in basal state and brain activation during visual erotic stimulation. As our results, we found the higher plasma concentration of sexual hormones was associated with the higher brain activation by erotic visual stimulation and prolactin and testosterone might have different target region.

In our result, brain processing to visual erotic stimuli was associated with significant activation in the limbic (hippocampal-amygdalar complex) and paralimbic regions (frontal and parietal lobe, parahippocampal gyri, insula), associative cortices (temporal and occipital cortices), thalamus, midbrain, pons and cerebellum. As expected, this result is consistent to finding of the previous studies.^20^,^23^,^26^,^27^ As regards the neurobehavioral model of the brain related in sexual arousal proposed by Redouté et al.^20^ composed of the cognitive, emotional, motivational, and autonomic component, each component includes several areas having a same functional meaning. The cognitive component includes the frontal and parietal lobe, occipitotemporal cortex, and cerebellum. Activation of the amygdale and insula, the caudal part of the anterior cingulate gyrus and nucleus accumbens belong to the emotional component and the motivational component, respectively. Finally, the autonomic component includes the hypothalamus and the rostral part of the anterior cingulate gyrus. In the result of the current study, the visual erotic stimulation induced brain activation included all components consisting cognitive, emotional, motivational and autonomic ones proposed by Redouté et al.^20^ (supplementary Table 1) This means that erotic stimuli used in our study was enough, at least, to induce attention as sexual incentives.

Our regression analysis showed that the concentration of plasma prolactin in basal state has a positive association the brain activity for the visual erotic stimulation in several regions, including left middle frontal gyrus, paracingulate/superior frontal/anterior cingulate gyri, the bilateral parietal lobule, the right cerebellum. Interestingly, these areas belong to a part of cognitive component of Redoute's neurobehavioral model of sexual arousal. Therefore, we propose that the plasma concentration of prolactin in basal state is positively correlated to the cognitive component for sexual arousal. Most of the regions overlapped with the regions positively correlated with subjective sexual desire to erotic pictures (Table 1, Figure 1C and D). However, the negative result of the correlation analysis suggests the concentration of prolactin in basal state may not be directly related with subjective sexual desire to erotic pictures. In terms of hyperprolactinemia induced sexual dysfunction, our result, the positive correlation between prolactin and the activity of cognitive component for sexual arousal finding, may look like an inconsistent finding. Some studies, however, suggested acute or short-term elevation of prolactin might affect the facilitatory effect to sexual behavior, though the limited evidences in rat.^32^,^33^ Thus, it is considered that concentration of prolacin in our study was in the range of normal level, and duration of hyperprolactinemia, even if abnormally high level of prolactin.

The plasma concentration of testosterone in basal state showed a statistically significant positive association with the activity of the bilateral supplementary motor area (SMA) in our results. The SMA is known as a key structure for behavioral planning and execution. Both human and primates studies have reported an importance of SMA in motor tasks that demand retrieval of motor memory. The SMA appears also crucial in temporal organization of movements,^34^,^35^ and more active when performing a sequence already learned, while pre-SMA is involved in acquiring new sequences.^36^,^37^ Thus, the SMA has been suggested to be a motor-limbic interface in the transformation of emotional experiences into motor actions including erectile responses.^38^,^39^ Transforming of erotic visual inputs into the related goal-directed behavior, it is showed that the SMA may be regarded as the motivational component of Redoute's model.^20^ Therefore, our result suggests that testosterone in basal state associated with motivational aspect for sexual response. Our finding is consisted with previous study^18^ showed correlation between testosterone and sexual motivation, and might be showed this result in brain aspect with neuroimaging study.

Although the precise mechanism cannot be explained with the results of the current study, we consider possible mechanisms of prolactin in brain. The one possibility is direct effect of prolactin. The prolactin-receptor mRNA expressed on several cerebral areas suggests the possibility of the direct effect of prolactin in central nervous system.^40^ Several reports demonstrated that prolactin-receptor mRNA were expressed to varying degrees in the cerebral cortex as well as choroid plexus, preoptic area, mediobasal hypothalamus, amygdale, pons-medulla in both male and female rats.^41^,^42^ Although prolactin can not pass the blood-brain barrier because of its size of 199 amino acid peptide, it may reach the cortical areas through the choroid plexi. The other possibility is the indirect effect of prolactin through the dopamine system. Prolactin may feedback to dopaminergic system which is related the pleasure such as sexual behavior. An example of this feedback is the surge of release of prolactin in orgasmic state.^43^ If this feedback also works in basal state, the plasma concentration of prolactin may be related with the activity in dopaminergic, such as incerto-hypothalamic, mesolimbocortical or nigrostriatal, pathway^44^ (e.g., a person with the higher dopaminergic activity may have the higher prolactin level in basal state). The prolactin level in basal state may affect the activity of cortical region, medial frontal and anterior cingulate cortex of the cognitive component, for erotic stimulation through dopaminergic pathway such as mesolibocortical dopaminergic system which originated in the ventral tegmental area and projects to the medial limbic system including mesial frontal cortex. The association of prolactin with the brain activity in the other area of cognitive component such as middle frontal, superior parietal cortex may a secondary phenomenon which reflects the interaction between prolactin and dopaminergic activity in incerto-hypothalamic, mesolimbocortical or nigrostriatal pathway. The testosterone in basal state may also associate with the activity of SMA directly or indirectly through the interaction with dopamine in nigrostriatal or mesolibocortical pathway. However, further studies are needed to confirm the direct or indirect effect of sexual hormones such as prolactin, testosterone on a specific dopaminergic pathway and to explore the possible mechanism of the effect.

The hormones having our attention, prolactin and testosterone, have a circadian periodicity, and it might be a confounding factor in our study. Concentration of prolactin in adult men is the higher at night than the day, but it is relatively consistent in the daytime.^45^,^46^ The diurnal variation of the plasma concentration of testosterone is reported that the highest in the morning and gradually decreased across daytime in adult men.^29^,^30^ Our correlation analyses were failed to show any relationship between the scan time, corresponded to the time for blood sampling, and the plasma concentration of the two sexual hormones. To decrease of the confounding effect of the diurnal variation, the time of blood sampling for each subject should be same. In spite of the limitation of our study, our results suggested inter-subject variation of the plasma concentration of testosterone and prolactin might associate with brain activity.

Our result show no significant correlation between the plasma concentration of testosterone and subjective sexual desire raise the question whether the plasma concentration of testosterone in basal state is associated with sexual behavior. One possible reason is relatively small sample size for performing correlation analysis. Another is subjects would not report their feeling as much as they felt because they were not informed what exactly were viewed and/or their cultural background. The result of the current study showed the positive correlation of subjective feeling to erotic pictures with the brain activity of the cognitive, not emotional or motivational, component. Thus, the other is subjects would report the degree of attentional incentives, not sexual arousal or drive, as their subjective feeling.

Complex neuroendocrine system for sexual response in human might not be explained with just two sexual hormones, prolactin and testosterone. Other hormones, such as oxytocin or estrogen, and neurotransmitters, such as serotonin, norepinephrine could be related sexual behavior. Oxytocin, especially, is increased during warm social contact with the partner, such as hugging,^47^ and is related to monogamous pair bonding, social cognition of mating partner and sexual interaction.^48^ The fMRI study for maternal or romantic love showed activation of the reward system including the anterior cingulate cortex, putamen, caudate nucleus, periaqueductal gray, and substantia nigra.^49^ These regions coincide with areas rich in oxytocin receptors.^50^ These previous fMR studies suggest that oxytocin may be related with limbic area among rewarding component and/or emotional component. Further studies are needed to explore the relationship between the erotic stimulation related-brain activity and oxytocin.

### Limitations of the study

In our knowledge, the current study is the first fMR study presented the association of sexual hormones in basal state with brain activity for visual erotic stimuli. However, it has a several methodological and interpretational limitations. We were not able to show a significant activation of the hypothalamus that is correlated to penile tumescence^23^ or sexual intensity^51^ to erotic stimuli, as previously reported by other fMRI studies of sexual arousal. The one possible reason is a relatively small sample size of 12 subjects. The other is that the presented erotic images might not be enough to induce the sexual arousal such as penile response. Three times repetition of the same condition block in our experimental design might be problematic if a certain brain activity sustained across the 3 blocks. This activity might not be separated from various fMR noises using high pass filter. We considered this issue. We, however, have believed the possible influence of erotic block to the subsequent happy-faced block could be more problematic than that of the repetition of the same condition block like our experimental design. Our results from the ERO minus HA contrast indicated that the induction of attention as sexual incentives was achieved with our study paradigm. In terms of diagnostic exclusion, Structured Clinical Interview for DSM-IV would be better than DSM-IV. Likert scale of 8, not 5 or 7, would affect mean score. However, the type of Likert scale may not affect the regression analyses because of their demeaned scale. Only Korean male subjects were included in the study, so this factor maybe affects the generalization of our results. Women may be show the different result from men because of the effect of estrogen on sexual behavior. Further studies might be needed to explore the relationship between sexual hormone including estrogen and brain activity in women and ethical or cultural difference in sexual behavior.

Considering the above statements and our finding, prolactin and testosterone may have a specific target regions associated with sexual response, cognitive and motivational components, respectively. Our study could be applied to explore the hormonal effect on gender difference in sexual behavior, the specific functional component of sexual dysfunction and any hormone-brain relationship using functional neuroimaging.

## Figures and Tables

**TABLE 1:**
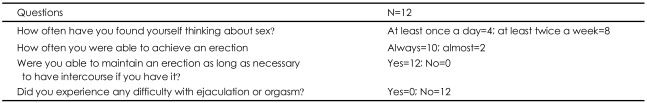
Subjects' sexual activities and sexual function including sexual desire, ability to have an erection, maintenance of erection, ejaculation failure during the past week

**TABLE 2:**
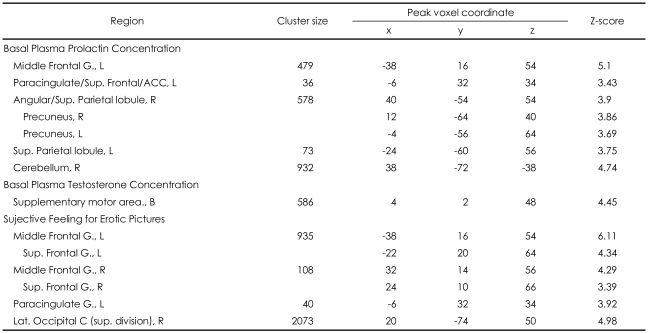
Regions showed the association of plasma concentration of sexual hormones in basal state with brain activation for erotic pictures and regions showed the correlation between subjective sexual desire for erotic pictures and brain activation for erotic pictures

L: left, R: right, B: bilateral, G: gyrus, C: cortex, Lat: lateral, Sup: superior, ACC: anterior cingulate gyrus. The coordinates of maximally activated voxels are given in MNI space. All activations identified at cluster-level significance of p<0.05 (corrected) for a spatial extent of at least 10 voxels

## Supplementary Material

SUPPLEMENTAL TABLE 1 Brain regions activated for the contrast of sexual arousal versus happy conditions
